# Evaluation of an Intervention to Improve Quality of Single-best Answer Multiple-choice Questions

**DOI:** 10.5811/westjem.2018.11.39805

**Published:** 2018-12-03

**Authors:** Kevin R. Scott, Andrew M. King, Molly K. Estes, Lauren W. Conlon, Jonathan S. Jones, Andrew W. Phillips

**Affiliations:** * Perelman School of Medicine at the University of Pennsylvania, Department of Emergency Medicine, Philadelphia, Pennsylvania; † The Ohio State University Wexner Medical Center, Department of Emergency Medicine, Columbus, Ohio; ‡ Loma Linda University Medical Center, Department of Emergency Medicine, Loma Linda, California; § Merit Health Central, Department of Emergency Medicine, Jackson, Mississippi; ¶ University of North Carolina, Department of Emergency Medicine, Chapel Hill, North Carolina

## Abstract

**Introduction:**

Despite the ubiquity of single-best answer multiple-choice questions (MCQ) in assessments throughout medical education, question writers often receive little to no formal training, potentially decreasing the validity of assessments. While lengthy training opportunities in item writing exist, the availability of brief interventions is limited.

**Methods:**

We developed and performed an initial validation of an item-quality assessment tool and measured the impact of a brief educational intervention on the quality of single-best answer MCQs.

**Results:**

The item-quality assessment tool demonstrated moderate internal structure evidence when applied to the 20 practice questions (κ=.671, p<.001) and excellent internal structure when applied to the true dataset (κ=0.904, p<.001). Quality scale scores for pre-intervention questions ranged from 2–6 with a mean ± standard deviation (SD) of 3.79 ± 1.23, while post-intervention scores ranged from 4–6 with a mean ± SD of 5.42 ± 0.69. The post-intervention scores were significantly higher than the pre-intervention scores, *x*^2^(1) =38, p <0.001.

**Conclusion:**

Our study demonstrated short-term improvement in single-best answer MCQ writing quality after a brief, open-access lecture, as measured by a simple, novel, grading rubric with reasonable validity evidence.

## INTRODUCTION

The use of single-best answer multiple-choice questions (MCQ) in examinations is ubiquitous in medical education. Although guidelines for writing MCQs exist, item writers often receive little to no formal training, potentially reducing the validity of examinations by introducing construct-irrelevant variance.^1^–^3^ Extended educational interventions in the area of item writing have been shown to improve written item quality with shorter interventions showing a similar impact.^4^–^6^ The literature suggests learners involved in item writing find it to be a positive learning experience that potentially improves performance on a summative assessment.^7^–^10^

The National Board of Medical Examiners (NBME) provides both a detailed, open-access guide for exam- question writing and an online training module.^11^–^13^ These tools provide instruction for writing high quality MCQs and are used in the design of basic and clinical science exams, but they are lengthy and oriented toward experienced question writers. Other tools remain lengthy and either require in-person workshops or are designed for self-study and require a prerequisite of basic question-writing understanding. Additionally, the literature lacks a simple MCQ quality metric with strong validity evidence. The two objectives of this study were to 1) establish validity evidence for a novel MCQ evaluation tool, and 2) evaluate the efficacy of a brief didactic lecture on MCQ question writing.

## METHODS

### Study Setting and Participants

We sought student and resident volunteers from the American Academy of Emergency Medicine Resident and Student Association, and conducted the educational intervention in September 2017. The study was granted exemption status by the University of Pennsylvania Institutional Review Board.

### Multiple-choice Question Quality Assessment Tool Derivation

We created a MCQ quality assessment tool based on expert opinions (AWP, KRS, JJ) of the most important components contained in the question-writing lecture; it is based on multiple, well-accepted sources, supporting content evidence.^11^,^12^ Two of the experts have formal education backgrounds including master’s degrees (AWP and KRS) that included advanced training in item writing and quality assessment. The third expert (JJ) has taught question writing for several years to national audiences. We followed current standards that endorse validity based on Messick’s model.^14^–^16^ We created six items, each rated on a binary “present” or “not present” scoring system with a total minimum potential scale score of zero and a maximum potential scale score of six (Figure). Two additional educators (AK and ME) reviewed the rubric and shared their interpretations, which were aligned with the item objectives, supporting response-process evidence. A set of 20 questions with intentional errors was created (AWP), available in Appendix A, for the initial validity evidence assessment.

### Training Module Creation and Assessment of Impact

The training module was created by an item-writing expert (JJ) using PowerPoint (Microsoft Corporation, Redmond, WA) with recorded voice-over (iMovie, Apple Inc., Cupertino, CA), allowing for independent completion by learners. The training module itself has been previously published in an open- access curriculum database and was based on principles of item writing as described by the NBME.^4^,^11^,^12^,^17^ Participants were asked to write three novel, single-best answer MCQs based on a two-page excerpt from an emergency medicine board review textbook about trauma just prior to the lecture. They then watched the question-writing lecture together on YouTube (Google Inc., Mountain View, CA) on a conference call followed by a 10-minute question and answer period with a question-writing expert different than the lecturer (AWP). Participants were then asked to write three new, single-best answer MCQs based on the same excerpt immediately after the lecture.

Pre- and post-intervention MCQ quality scores were determined by two item-writing experts (AK, ME) via the item quality assessment tool. Discrepancies were decided by a third item-writing expert (LC).

### Statistical Analysis

We first performed descriptive summaries including mean and standard deviation (SD), frequencies, and total responses. Internal reliability was assessed using Cohen’s kappa. We decided a priori to compare pre- and post-lecture scores using the non-parametric Friedman’s analysis of variance (ANOVA), given the expected range to be relatively small and low likelihood of having an even distribution of the standard error of the mean. Friedman’s ANOVA is essentially a non-parametric, repeated measures one-way ANOVA. A p-value less than 0.05 was considered statistically significant. We performed all analyses using SPSS version 24 (IBM Corporation, Armonk, NY).

## RESULTS

### Multiple-choice Question Quality Assessment Tool Validity Evidence

The internal structure evidence was moderate when the tool was applied to the 20 practice questions (κ=.671, p<.001). The tool demonstrated excellent internal structure when applied to the true dataset of questions created by the students and residents (κ=0.904, p<.001) with only eight discrepancies in 264 cases (48 total requested questions – 4 missing questions = 44 total questions with 6 points each yielding 264 cases), evaluated by two different researchers. Evidence of consequence was demonstrated as part of the other primary objective of this study, in which pre- and post-lecture scores were different. As this was a stand-alone study, we were unable to evaluate for relationships with other variables.

### Training Module Impact on Item Quality

A total of eight residents and students consented and participated in the lesson, of whom seven provided both pre- and post-lecture MCQs. One participant provided two pre-lecture questions rather than three, and another provided no post-lecture questions, thus totaling four total missing questions of the 48 possible total questions (8 × 3 × 2). Missing questions were excluded pairwise since the post questions were edits of the original questions; therefore, four missing questions led to elimination of eight total questions. We analyzed a total of 40 questions (20 pre- and 20 post-lecture). The MCQ quality scale scores for pre-intervention questions written by the learners ranged from 2 - 6 with a mean ± SD of 3.79 ± 1.23, while post-intervention scores ranged from 4 – 6. The post-intervention scores were significantly higher than the pre-intervention scores, *x*^2^(1) =38, p <0.001.

## DISCUSSION

The current study supports the efficacy of a short, high-yield lecture to teach best evidence in developing single-best answer MCQs. The study also provides strong validity evidence for a novel tool by which to evaluate the structure of single-best answer MCQs.

Although multiple prior studies have evaluated outcomes from an educational intervention to improve MCQ writing, the current study is the first available remotely, free to the public, and at approximately 30 minutes in length is the shortest.^5^,^6^,^18^ These differences are important because this efficacious education intervention is replicable in any setting, whereas in-person workshops may vary with the instructor, size of the group, and other factors. The open-access availability through the educational platform at the *Journal of Education and Teaching in Emergency Medicine* (JETem) and its brief duration provide a practical advantage to this educational intervention as well.^17^ Future work should directly compare other tools against this one.

Another important contrast to prior studies is the target group. Much focus has been placed on faculty development, yet educators are seeing the benefits of learners writing questions.^5^–^9^,^18^–^21^ To this end, the current educational intervention was specifically designed for novice MCQ writers and tested in a sample of students and residents. It can be easily adopted by clerkship directors and program directors to use with students and residents as both a learning tool and as preparation to write questions as junior faculty members in future years.

This study lastly provides a checklist with reasonable validity evidence and strong inter-rater reliability when applied to the real-world questions. This is in contrast to other checklists that exist but are limited to content validity by experts.^18^ It is unclear why the instrument had better inter-rater reliability with the real questions than when applied to the sham questions. We suspect this finding simply uncovered the inherent limitation of sham tests in which the author was trying to elicit specific flags in the tool. The strong performance with the live questions is reassuring.

## LIMITATIONS

Our study must be interpreted in the context of several limitations. Most importantly, we studied a short-term outcome. This variable must be a precursor to follow-up, long-term learning outcomes to fully elucidate the efficacy of the intervention. It is also important to highlight that the intervention and assessment tool are intended to improve the structure of MCQs. Such proper practices are associated with good question quality as ascertained through psychometric analysis, but they are beyond the scope of our initial study. Additionally, our study recruited volunteers who may have been more motivated to improve their MCQ writing skills than students and residents in the general population. Finally, although the MCQ quality tool was applied against a test group of questions and a real-life group of questions, it was nonetheless a small sample of questions with a small number of participants, and the tool should be tested against more questions and more raters.

## CONCLUSION

Our study demonstrated short-term improvement in single-best answer MCQ writing quality after a brief, open-access lecture, as measured by a simple, novel, grading rubric with reasonable validity evidence.

## Supplementary Material



## Figures and Tables

**Figure:**
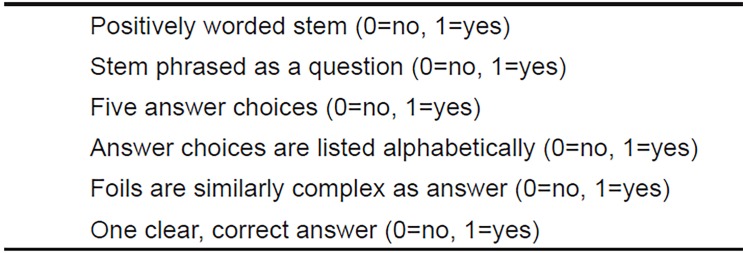
Multiple-choice questions quality assessment tool.
