# Supplementary material for: Evaluation of an Intervention to Improve Quality of Single-best Answer Multiple-choice Questions
Source: West J Emerg Med. 2018 Dec 3;20(1):11–4. doi: 10.5811/westjem.2018.11.39805 (PMC6324722; doi:10.5811/westjem.2018.11.39805)
Supplement: Supplementary file 1 [file wjem-20-11-s001.doc]

**Appendix A.**

1) A 22-year-old man was the restrained driver of an automobile involved in a head-on collision with a pick-up truck. His air bag deployed and he was ambulatory at the scene. He denies loss of consciousness or neurologic complaints. He has a history of hemophilia type A. His physical exam is remarkable only for a 1-cm laceration on his forehead, apparently where his head struck the rearview mirror. A head CT scan shows no evidence of acute intracranial injury or bleed. The most appropriate treatment includes administration of:

A. 12.5 units/kg of factor VIII, then discharge.

B. 50 units/kg of factor VIII, then discharge.

C. 50 units/kg of factor VIII, then admit to hospital for further observation.

D. 0.3 mcg/kg of desmopressin, then discharge.

E. 0.3 mcg/kg of desmopressin, then admission to hospital for further observation.

2) A 24-year-old woman presents to the emergency department after slashing her left wrist with a kitchen knife. She has a very superficial laceration, which spares the dermis. You note many well-healed linear scars on both wrists. When you look at old records, you find that she was admitted 2 months ago after an amoxicillin overdose. She explains that she just had an argument with her significant other and is sorry for hurting herself, but she is afraid he

will leave her. This presentation is most consistent with:

A. Borderline personality disorder.

B. Histrionic personality disorder.

C. Antisocial personality disorder

D. Malingering.

E. Manic disorder.

3) What is the most common x-ray finding in a patient with mycoplasma pneumonia?

A. Abscess.

B. Cavitations.

C. Interstitial, patchy infiltrates.

D. Large pleural effusion.

E. Mediastinal lymphadenopathy.

4) A 32-year-old man complains of fatigue, myalgia, and headache. He recently returned from a tour of East Africa, where he spent time camping and backpacking. His temperature is 39.2 C and his physical exam is normal. You send a thick and thin blood smear, which is positive for Plasmodium falciparum, with a parasitemia >10%. What is the next best step in management?

A. Give acetaminophen and send the man home for follow-up with the local public health district.

B. Give doxycycline and send the man home for follow up with his family doctor.

C. Give malarone and admit the patient to a non-monitored floor bed.

D. Give primaquine and admit the patient to a non-monitored floor bed.

E. Give intravenous quinidine and admit the patient to an intensive care unit.

5) A 90-year-old man with a history of heart failure, atrial fibrillation, and hypertension fell down the basement steps 4 hours ago. He takes 3 mg of warfarin each morning. His GCS score is 15, and he has good strength and coordination in all of his extremities. His INR is 4. A CT scan of the head shows a small intraparenchymal hemorrhage. His BP is 200/100 and his HR is 70 and regular. What is the best treatment for this patient?

A. Fresh frozen plasma (FFP) alone.

B. 10 mg of intravenous vitamin K and FFP.

C. 10 mg of intravenous vitamin K and weight-based prothrombin complex concentrates.

D. Recombinant factor VIIa.

E. 10 mg of intravenous vitamin K alone.

6) A 4-year-old girl presents with ascending flaccid paralysis and decreased deep tendon reflexes. She has had no preceding illnesses. The family just returned from a camping trip, and examination of the girl reveals a tick embedded in her scalp. Which action is most likely to provide benefit in her recovery?

A. Administering high-dose corticosteroids.

B. Administering intravenous immunoglobulins.

C. Intubating and providing respiratory supportive care.

D. Performing plasmapharesis.

E. Removing the tick.

7) Choose the correct statement about a prehospital disaster plan and its contents:

A. Most casualties are transported to a hospital over a relatively long time, with most patients arriving within 6 hours after the disaster has occurred.

B. Most patients presenting to an emergency department after a disaster have injuries that require advanced trauma services.

C. Physician training for mass casualty situations is usually a requirement for medical staff privileges.

D. The Joint Commission reviews only a hospital’s internal disaster plan.

8) A STEMI patient who received thrombolytic therapy 1 hour ago at a small rural hospital develops an accelerated idioventricular rhythm while awaiting transfer to a tertiary care facility. He is not experiencing chest pain and he is currently asymptomatic. What is the most appropriate management?

A. Lidocaine.

B. Atropine.

C. Observation.

D. Adenosine.

E. Immediate cardioversion.

9) After sustaining a direct blow to the larynx from an elbow during a basketball game, a high-school student now has rapidly developing dysphonia, stridor, hoarseness, and dyspnea. What is the next best step in management?

A. Give intravenous steroids 1mg/kg intravenously.

B. Obtain a CT noncontrast scan of the neck followed by contrast to evaluate for hematoma.

C. Intubate.

D. Call ENT to perform cricothyroidotomy at the bedside in the Emergency Department.

E. Obtain a portable x-ray AP and lateral view to evaluate the larynx.

10) A 75-year-old woman with a history of polymyalgia rheumatica has right-sided headache that has been worsening over the past month. The headache is predominantly in the right temporal area, and she says that her jaw hurts when she chews. Her vital signs are normal. You find focal tenderness over the right temporal area, but the remainder of her examination is normal. Her erythrocyte sedimentation rate (ESR) is 85 mm/hr. You should next:

A. Start an oral anti-inflammatory agent, discharge the patient, and refer her to a general surgeon for temporal artery biopsy.

B. Start parenteral steroids and arrange for temporal artery biopsy, either as inpatient or outpatient.

C. Order head CT with contrast to rule out aneurysm.

D. Start oral steroids and refer the patient for follow-up with her primary care provider.

11) Your hospital’s infection control nurse notifies you that the infant you admitted yesterday with meningitis is growing meningococcus from both blood and cerebrospinal fluid cultures. Who should receive prophylactic antibiotics?

A. Emergency department nursing personnel.

B. Personnel at the daycare center where the child spent 6 hours on Monday.

C. Paramedics who performed mouth-to-mouth resuscitation prior to intubating the child.

D. The physician who did the history and physical examination.

E. The resident who performed the spinal tap.

12) A 28-year-old woman is complaining of a panic attack. She has recently been having more frequent panic attacks associated with intermittent pounding headaches. She also reports palpitations and sweatiness during these episodes.

Her primary doctor diagnosed her with anxiety disorder and started her on an antidepressant but it hasn’t helped. Her vital signs are as follows: BP = 208/125, HR = 125, and T = 98.2 F. Other than being profoundly diaphoretic, the rest of her physical exam is normal. An ECG shows sinus tachycardia. All of the following are appropriate therapies EXCEPT:

A. Phentolamine and midazolam.

B. Lorazepam and haloperidol.

C. Phentolamine and propranolol.

D. Midazolam.

13) Which of the following statements concerning child abuse is true?

A. The reporting of child abuse to child protective services and/or the district attorney’s office is done at the discretion of the emergency physician.

B. Failure to report a case of child abuse may result in criminal prosecution as well as malpractice liability.

C. Physician-patient confidentiality takes precedence over the duty to report a case of child abuse.

D. In cases of suspected child abuse, the patient and/or parent may leave “against medical advice” as long as appropriate follow-up is arranged.

E. Immunity from civil or criminal liability is not guaranteed for good-faith reporting by mandatory or voluntary authorities.

14) What is the most common skin eruption caused by medication?

A. Bullae.

B. Macules.

C. Papules.

D. Striae.

E. Urticaria.

15) For the past week, a 22-year-old woman who uses IV heroin has experienced right-sided pleuritic chest pain associated with a dry cough, gradual exercise intolerance, weight loss, and diarrhea. Her vital signs are as follows: T = 38.2C, RR = 24, HR = 105, pulse ox = 86%, and BP = 105/67. On the pulmonary exam, you find generalized lymphadenopathy

and scattered rales bilaterally. Her chest x-ray shows no infiltrate and her blood work is significant for an elevated LDH. The organism most commonly associated with this disease is:

A. H. influenzae.

B. Herpes simplex.

C. Pneumocystis jiroveci.

D. Pseudomonas aeruginosa.

E. S. pneumoniae.

16) For the over-the-counter “morning after” pill to be maximally successful in preventing pregnancy after unprotected intercourse, it must be taken within:

A. 12 hours.

B. 24 hours.

C. 36 hours.

D. 72 hours.

E. 7 days.

17) What is most common complication of a tracheostomy?:

A. Infection.

B. Hemorrhage.

C. Accidental airway obstruction.

D. Accidental decannulation.

E. Aluminum toxicity.

18) A 35-year-old woman is brought to the ED by her husband, who says he found an empty bottle of amitriptyline at her bedside. The patient is unresponsive and shows signs of cyclic antidepressant toxicity. What is the next most appropriate step in management?

A. Administer sodium bicarbonate.

B. Administer benzodiazepines.

C. Give multiple-dose activated charcoal.

D. Intubate the patient.

19) A 23-year-old man became lost while hiking in the desert and hiked the last few miles after dark. During the last portion of his hike, he felt a sharp pain in his leg; when he got to his car, he saw two small puncture wounds. He does not complain of pain at the site; however, he is experiencing blurry vision. On exam, you note minimal redness and swelling at the site. You also note mild ptosis. The most likely source of his symptoms and initial management are:

A. Rattlesnake: incise the wound with suction, provide local wound care as well as supportive care, and administer antivenin.

B. Scorpion: compress the wound, immobilize the leg, provide local wound care as well as supportive care, and administer antivenin.

C. Coral snake: incise the wound with suction, provide local wound care as well as supportive care, and administer antivenin.

D. Coral snake: compress the wound, immobilize the leg, provide local wound care as well as supportive care, and

administer antivenin.

E. Scorpion: provide local wound care as well as supportive care and administer benzodiazepines and antivenin.

20) A 76-year-old man with a history of hypertension and multiple kidney stones presents with right flank pain radiating to the groin. A urine dipstick shows the presence of blood, leukocyte esterase, and nitrites. On physical examination, you find that he has mild mid-epigastric tenderness. His blood pressure is 90/60 with a heart rate of 117. Your first consideration for a diagnosis is:

A. Rupturing aortic aneurysm.

B. Urinary tract infection.

C. Renal colic.

D. Pancreatitis.

E. Testicular torsion.
